# Impact of partial volume correction on radiomics reproducibility in theranostic SPECT/CT imaging

**DOI:** 10.1002/mp.70427

**Published:** 2026-04-03

**Authors:** Mohammad Saber Azimi, Maryam Cheraghi, Mohammad Ali Ghodsi Rad, Mohadeseh Bayat, Maryam Alhashim, Habibollah Dadgar, Mahmoud Alabedi, Nahid Ibrahim, Enas Alwuhaib, Hossein Arabi, Arman Rahmim, Habib Zaidi

**Affiliations:** ^1^ Doctoral School of Applied Informatics and Applied Mathematics Óbuda University Budapest Hungary; ^2^ University Research and Innovation Center Óbuda University Budapest Hungary; ^3^ PET‐CT Unit, Department of Nuclear Medicine, Masih Daneshvari Hospital Shahid Beheshti University of Medical Sciences Tehran Iran; ^4^ Department of Nuclear Medicine, Shohada‐e Tajrish Medical Center School of Medicine Shahid Beheshti University of Medical Sciences Tehran Iran; ^5^ Department of Nuclear Medicine, Taleghani Hospital, School of Medicine Shahid Beheshti University of Medical Sciences Tehran Iran; ^6^ Department of Radiology, College of Medicine Imam Abdulrahman Bin Faisal University Dammam Saudi Arabia; ^7^ Medical Imaging Services Center King Fahad Specialist Hospital Dammam Dammam Saudi Arabia; ^8^ Cancer Research Center, RAZAVI Hospital Imam Reza International University Mashhad Iran; ^9^ Division of Nuclear Medicine & Molecular Imaging Geneva University Hospital Geneva Switzerland; ^10^ Departments of Radiology and Physics University of British Columbia Vancouver British Columbia Canada; ^11^ Department of Basic and Translational Research BC Cancer Research Institute Vancouver British Columbia Canada; ^12^ Department of Nuclear Medicine and Molecular Imaging University of Groningen Groningen Netherlands; ^13^ Department of Nuclear Medicine University of Southern Denmark Odense Denmark

**Keywords:** 177Lu SPECT/CT, liver metastasis, partial volume correction, quantitative SPECT, radiomics reproducibility, theranostics

## Abstract

**Background**: Radiomics has shown potential for quantitative characterization of tumors in molecular imaging; however, its clinical translation in theranostic ^1^ ^7^ ^7^Lu SPECT/CT remains limited due to poor robustness of extracted features to reconstruction variability and partial volume effects. Establishing reproducible radiomics biomarkers across correction strategies is therefore a prerequisite for reliable clinical modeling and treatment monitoring.

**Purpose**: This study aimed to evaluate radiomics feature reproducibility, defined as the stability of feature values across different partial volume correction (PVC) strategies and reconstruction settings, in clinical ^1^ ^7^ ^7^Lu SPECT/CT imaging. In addition, we explored two volumetric shape‐based indices, the metastasis‐to‐liver ratio (MLR) and metastasis‐to‐spare liver ratio (MSLR), as surrogate markers of hepatic metastatic burden in the theranostic treatment setting.

**Methods**: In 13 patients (40 scans) treated with ^177^Lu, 837 radiomics features were extracted from 11 abdominal regions and metastases on SPECT/CT using original and wavelet‐decomposed images across four bin widths (50–200). Two post‐reconstruction PVC methods, namely Richardson‐Lucy (RL) and Reblurred Van Cittert (RVC), were applied. Feature reproducibility was quantified using two complementary metrics: the intraclass correlation coefficient (ICC) to assess feature‐level stability across PVC strategies, and the concordance correlation coefficient (CCC) to evaluate pairwise agreement and systematic bias among reconstruction methods. Visual image quality assessments were independently performed by two experienced nuclear medicine specialists in a blinded setting. Exploratory metastatic tumor burden was assessed descriptively using 3D shape‐based MLR and MSLR indices.

**Results**: Low‐frequency wavelet decomposition (LLL‐wavelet) and original features showed the highest reproducibility (ICC ≥ 0.90 in >95% of liver and metastasis features at BW50), whereas high‐frequency features and larger bin widths demonstrated reduced stability. CCC analysis revealed excellent agreement between RL and RVC (≥0.95 in major organs at BW50–100), while agreement with uncorrected SPECT (no PVC) was consistently lower, especially for high‐frequency features. RL achieved higher visual scores in sharpness and contrast (*p* < 0.01), with good inter‐reader agreement supporting the consistency of these assessments. MLR/MSLR demonstrated inter‐patient variability and were explored descriptively as indices of metastatic liver burden.

**Conclusions**: Reproducibility in theranostic SPECT radiomics is highly feature‐ and organ‐dependent and is further influenced by scanner‐specific factors and reconstruction protocols, which remain critical for real‐world clinical translation. RL and RVC showed stronger mutual agreements than each with uncorrected SPECT. Importantly, only RVC translated visual improvements into enhanced feature‐level reproducibility, while RL provided the most consistent overall balance of reproducibility and image quality, supporting its role as the preferred PVC strategy for clinical and modeling applications. Robust radiomics feature selection as well as standardized reproducible PVC strategies are essential to generate methodological harmonization for future clinical translation and to support integration of radiomics analyses into personalized SPECT theranostics.

## 1 INTRODUCTION

Radiomics enables the extraction of high‐dimensional quantitative features from medical images for predictive modeling in precision oncology.^1^, ^2^, ^3^, ^4^ While widely studied in PET and CT, its application to post‐therapy ^1^ ^7^ ^7^Lu SPECT/CT is less explored, though recent studies show promise for clinical decision support in radionuclide therapy.^5^, ^6^ ^1^ ^7^ ^7^Lu‐labeled agents, such as [^1^ ^7^ ^7^Lu]Lu‐DOTATATE and [^1^ ^7^ ^7^Lu]Lu‐PSMA are standard for treating neuroendocrine and prostate cancers.^7^, ^8^ Theranostic SPECT/CT imaging plays a crucial role in dosimetry calculation, therapy monitoring, and lesion characterization.^9^, ^10^, ^11^ However, several technical limitations, most notably low spatial resolution,^12^, ^13^ partial volume effects (PVE),^14^, ^15^ and image noise,^16^ can significantly affect the stability and interpretability of radiomics features extracted from these images. Partial volume correction (PVC) techniques have been developed to mitigate PVE, especially in small lesions or organs with heterogeneous uptake.^17^ While existing studies have applied PVC methods to improve quantitative accuracy in ^177^Lu SPECT/CT, most efforts have focused on recovery coefficients and dosimetry enhancement.^18^, ^19^, ^20^, ^21^ Although PVC has mainly been used to improve uptake quantification, recent work has also begun to explore its influence on radiomics feature robustness, particularly in PET imaging (e.g., Azimi et al.^22^), whereas reproducibility‐oriented studies in post‐therapy ^1^ ^7^ ^7^Lu SPECT/CT remain scarce. To date, there is a lack of comprehensive evaluation of how PVC impacts the reproducibility of radiomics features, posing a challenge for their integration into reliable clinical models for treatment planning and response assessment. Radiomics feature reproducibility is defined as the consistency of extracted features across varying image processing pipelines, such as reconstruction and PVC strategies, rather than scan–rescan repeatability under identical acquisition protocols.^23^ Feature reproducibility is therefore a key methodological metric of merit in radiomics, as it defines whether extracted biomarkers reflect true biological information or are primarily driven by technical processing variability.^24^ Importantly, establishing such robustness is a necessary prerequisite before radiomics biomarkers can be reliably used in downstream predictive modeling or clinical translation.^25^ However, reproducibility in theranostic ^1^ ^7^ ^7^Lu SPECT/CT remains insufficiently characterized, particularly in the presence of noise, resolution limits, and PVE.

Beyond reproducibility, clinical integration of SPECT/CT radiomics, particularly for treatment monitoring, requires stable, biologically meaningful biomarkers.^26^, ^27^, ^28^, ^29^ Volumetric indices, such as metastasis‐to‐liver ratio (MLR) and metastasis‐to‐spare liver ratio (MSLR) have shown promise in assessing intrahepatic tumor burden during therapy.^30^, ^31^ While these metrics have been used descriptively in PET‐based studies,^32^, ^33^, ^34^ their application in ^177^Lu SPECT/CT remains largely unexplored. Incorporating such indices alongside radiomics reproducibility analysis may enhance theranostics interpretation by bridging the gap between reproducible image features and clinically meaningful biomarkers of therapeutic response.

Reproducibility remains a major barrier to the clinical translation of radiomics.^35^ Factors, such as low resolution, image noise, segmentation variability, and preprocessing inconsistencies, can significantly impact feature robustness.^25^, ^36^, ^37^ Previous studies suggested that only 50%–60% of radiomics features in SPECT and PET meet acceptable reproducibility thresholds.^38^, ^39^ To ensure stability, it is crucial to standardize segmentation and correction pipelines.^40^, ^41^ In this study, we restrict analysis to non‐shape radiomics features and apply consistent volumes of interest (VOIs) across reconstruction methods to minimize inter‐procedure variability. Feature robustness is evaluated using both the Intraclass Correlation Coefficient (ICC) and Concordance Correlation Coefficient (CCC), enabling a comprehensive assessment at both feature and organ levels.^42^ To complement these analyses, inter‐rater reproducibility was also evaluated by comparing results of segmentations obtained from two independent nuclear medicine specialists.

This work provides a novel and systematic evaluation of radiomics feature reproducibility in theranostic ^177^Lu SPECT/CT, focusing on both abdominal organs and liver metastases. We apply two available post‐reconstruction PVC techniques, namely Richardson‐Lucy (RL) and Reblurred Van Cittert (RVC), using four predefined bin width discretization settings to assess their effect on reproducibility. A blinded, independent visual assessment by two experienced nuclear medicine specialists was used to evaluate perceptual image quality across the different methods. Additionally, to link reproducibility to clinical application, we explored theranostics volumetric biomarkers (MLR and MSLR) as exploratory volumetric indices of metastatic liver burden in the theranostic treatment setting. Through this work, we aim to identify reproducible features and correction strategies that support future development of reliable SPECT radiomics workflows for clinical research and theranostic applications.

## 2 MATERIALS AND METHODS

**FIGURE 1:**
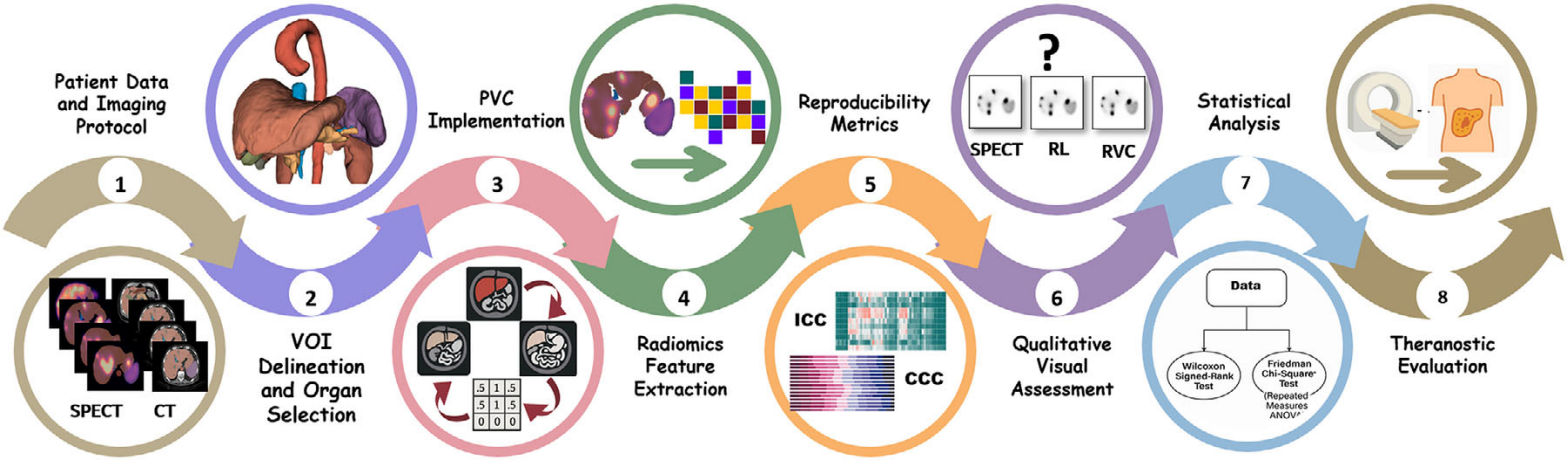
The overall study workflow for evaluating radiomics reproducibility and theranostics potential in theranostic ^177^Lu SPECT/CT imaging. The process begins with patient data acquisition and standardized SPECT/CT imaging (Step 1), followed by semi‐automatic segmentation of key organs and lesions (Step 2). Next, two partial volume correction (PVC) methods, Richardson‐Lucy (RL) and Reblurred Van Cittert (RVC), are applied alongside uncorrected SPECT images (no PVC) (Step 3). Radiomics features are then extracted from each organ and processing method (Step 4), and feature reproducibility is quantified using Intraclass Correlation Coefficient (ICC) and Concordance Correlation Coefficient (CCC) (Step 5). Two board‐certified nuclear medicine specialists independently performed blinded visual quality scoring of the reconstructed images using eight qualitative criteria on a 6‐point Likert scale (Step 6). Non‐parametric statistical tests—including the Friedman test and Wilcoxon signed‐rank test—were applied separately for each reader to compare reconstruction methods. Inter‐reader agreement was further assessed using Spearman's rank correlation and Cohen's weighted Kappa metrics (Step 7). Finally, an exploratory theranostics evaluation is conducted by calculating tumor‐to‐liver burden ratios and assessing radiomics‐driven malignancy indices across different treatments and timepoints (Step 8).

Figure 1 displays the overall workflow of the study protocol adopted to evaluate radiomics reproducibility when applying different PVC techniques to clinical ^177^Lu SPECT studies.

### 2.1 Patient data and imaging protocol

A total of 40^177^Lu SPECT/CT scans were retrospectively collected from 13 male patients undergoing either ^177^Lu‐PSMA‐617 or ^177^Lu‐DOTATATE therapy at the Medical Imaging Department, King Fahad Specialist Hospital—Dammam, Saudi Arabia. The patients were diagnosed with neuroendocrine tumors (NETs) or prostate cancer, according to the type of radioligand used. The age of the patients ranged from 37 to 76 years, with a mean ± standard deviation of 60.4 ± 10.4 years. Each patient underwent between one and four post‐treatment SPECT/CT scans during their therapy cycles. All SPECT/CT scans were acquired approximately 24 h after intravenous administration of ^177^Lu‐labeled compounds, with administered activities ranging from 6.92 to 8.14 GBq. Imaging was performed on a Symbia Intevo SPECT/CT system (Siemens Healthcare) using the following acquisition parameters:

- Medium‐energy low penetration (MELP) collimators
- 3 bed positions covering vertex to mid‐thigh
- 120 projections over 360° (10 s per projection)
- Body‐contouring (non‐circular) orbit
- 128 × 128 matrix, 4.8 × 4.8 mm pixel size

Immediately following SPECT acquisition, an unenhanced low‐dose CT scan was acquired for attenuation correction and anatomical localization. SPECT data were reconstructed using an iterative ordered‐subset expectation maximization (OSEM) algorithm (Flash 3D) with 4 iterations and 8 subsets. The reconstruction protocol included CT‐based attenuation correction, dual‐energy‐window scatter correction, collimator response modeling (resolution recovery) and quantitative calibration for conversion to standardized uptake values (SUVs). All patient data were anonymized before analysis. The study was approved by the local Institutional Review Board (IRB #RAD0343). The requirement for informed consent was waived due to the retrospective nature of the study and the use of anonymized data.

### 2.2 VOI delineation and organ selection

VOI delineation was performed semi‐automatically using the MONAI (Medical Open Network for AI) segmentation module,^43^ specifically the “Abdominal Organs TS1” model, implemented within 3D Slicer (version 5.8.0). The initial automatic segmentation included the following ten abdominal structures: aorta, gallbladder, inferior vena cava (IVC), left and right kidneys, liver, pancreas, spleen, stomach, portal vein and splenic vein.

Following the automated step, all ROIs were manually corrected slice‐by‐slice in the axial CT view by a trained medical image analyst to ensure anatomical accuracy. Special care was taken to adjust organ boundaries near areas of low contrast or adjacent to pathological uptake.

**FIGURE 2:**
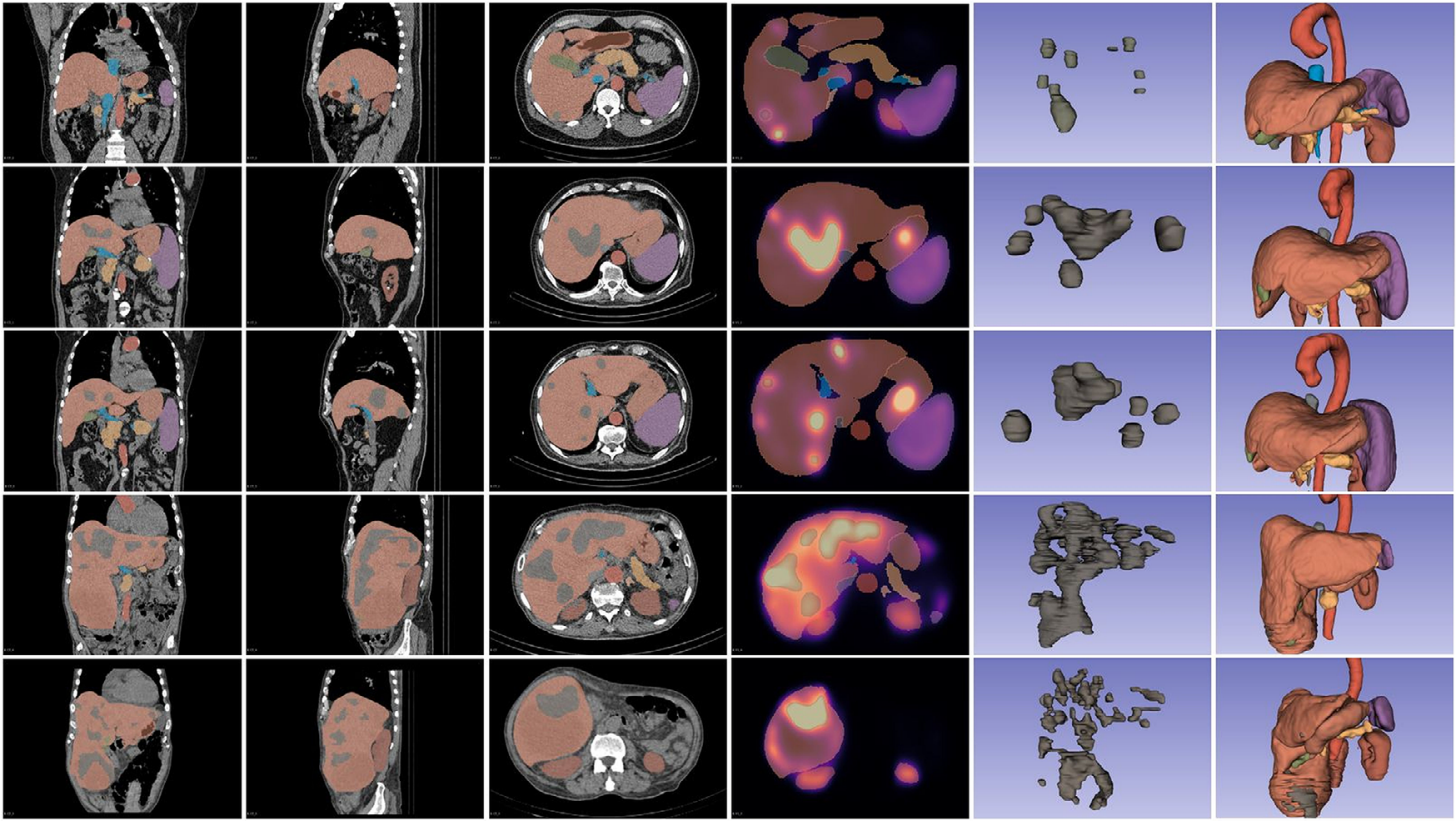
Representative segmentation outputs across 40 patients. Columns from left to right: multi‐planar reformatted CT views (coronal, sagittal, axial) with color‐coded VOIs for abdominal organs; SPECT with overlaid segmentations; 3D rendering of hepatic metastases; and 3D visualization of segmented abdominal anatomy including aorta, liver, gallbladder, kidneys, spleen, pancreas, stomach, and vasculature. Segmentations were initially obtained using the MONAI “Abdominal Organs TS1” model and manually corrected for anatomical accuracy. Liver metastases were delineated by a nuclear medicine specialist based on SPECT uptake and CT anatomical correlation.

In addition to anatomical organ segmentation, soft tissue metastases in the liver were manually contoured by a nuclear medicine specialist with 15 years of experience, based on focal uptake seen on the SPECT images and correlated with anatomical localization from CT. To further evaluate inter‐rater variability, a second nuclear medicine specialist (with 3 years of experience) independently performed the same segmentation procedure using the same software. Thus, each image had two sets of lesion masks from two raters. Only hepatic lesions that were clearly distinguishable from physiological background uptake were included in the analysis. To ensure consistency and reproducibility across the dataset, a standardized segmentation protocol was followed for all patients and scans. The segmentation process and quality control were performed blinded to the clinical outcome data. Representative examples of the segmentation and organ selection process are illustrated in Figure 2.

### 2.3 PVC implementation

To mitigate the impact of PVE in SPECT images, the PETPVC toolbox (version 1.2.11),^17^ developed in C++ using the Insight Segmentation and Registration Toolkit (ITK), was utilized. In this study, two established deconvolution‐based PVC methods were implemented, namely RL and RVC. These techniques operate directly on reconstructed SPECT images and do not require anatomical masks, making them suitable for image‐based correction workflows. Both methods rely on the system's point spread function (PSF) to iteratively deconvolve image blurring and restore spatial resolution. The PSF was modeled as a Gaussian function with a nominal full‐width at half‐maximum (FWHM) of 7.4 mm, based on manufacturer specifications under standard phantom measurement conditions. We note that effective clinical resolution may vary with collimator‐to‐patient distance and patient‐specific geometry. A Gaussian kernel with matching FWHM was used under the assumption of shift‐invariant PSF. Both RL and RVC methods were applied using 10 iterations, representing a commonly used compromise between resolution recovery (RR) and noise amplification in iterative deconvolution‐based PVC, as reported in previous quantitative imaging studies.^44^, ^45^

### 2.4 Radiomics feature extraction

A total of 837 radiomics features were extracted from both non‐PVC and PVC‐corrected SPECT images across 11 abdominal organ regions (as defined in the segmentation protocol), using the open‐source Pyradiomics Python package.^46^ The extracted features were categorized into six main feature classes:

- First‐Order Statistics (18 features)
- Gray Level Co‐occurrence Matrix (GLCM) (24 features)
- Gray Level Run Length Matrix (GLRLM) (16 features)
- Gray Level Size Zone Matrix (GLSZM) (16 features)
- Gray Level Dependence Matrix (GLDM) (14 features)
- Neighborhood Gray‐Tone Difference Matrix (NGTDM) (5 features)

These features were calculated from both the original intensity images and the eight wavelet‐transformed image types (LLH, LHL, LHH, HLL, HLH, HHL, HHH, and LLL), which are commonly used for multiscale texture characterization in radiomics,^47^, ^48^ resulting in a total of 837 features per scan. For intensity discretization, we employed a Fixed Bin Number (FBN) approach, following the recommendations of the Image Biomarker Standardization Initiative (IBSI).^49^ Four different bin numbers were evaluated: 50, 100, 150, and 200, to assess the effect of discretization on feature robustness and reproducibility.

### 2.5 Reproducibility metrics

To assess radiomics feature reproducibility under different correction strategies, the ICC^50^ and CCC^51^ were computed for each feature extracted from non‐PVC and PVC‐corrected images. ICC was calculated using a two‐way random‐effects model with absolute agreement [ICC(2,1)] following Koo and Li's guidelines.^52^ The ICC formula was:

(1) $$ICC=\frac{MSBS-MSE}{MSBS+\left(K-1\right)*MSE+\left(\frac{k}{n}\right)*\left(MSBM-MSE\right)}$$

where MSBS is the mean square between subjects, reflecting the variance of extracted radiomic feature values across different patients (or scans), as derived from the variance decomposition model used in ICC (2,1) computation. MSE is the mean square error (within subjects), MSBM is the mean square between measurements, *k* is the number of repeated measurements (e.g., PVC methods), and *n* is the number of subjects (e.g., organ‐level measurements).

Interpretation thresholds were:

- Excellent reproducibility: ICC ≥ 0.90
- Good reproducibility: 0.75 ≤ ICC < 0.90
- Moderate reproducibility: 0.50 ≤ ICC < 0.75
- Poor reproducibility: ICC < 0.50

The CCC was used to quantify agreement between two strategies by incorporating both precision and accuracy, offering a more stringent reproducibility metric than ICC. Unlike ICC, CCC accounts for systematic bias by evaluating deviation from the identity line (y=x). It was also used to assess inter‐rater agreement between segmentations from two independent raters. CCC was defined as:

(2) $$\rho_{c}=\frac{2\;Cov\;\left(x,y\right)}{\sigma_{x}^{2}+\sigma_{y}^{2}+{\left(\mu_{x}-\mu_{y}\right)}^{2}}$$

where $\mu_{x}$​ and $\mu_{y}$​ denote the means of variables x and y, $\sigma_{x}^{2}$ and $\sigma_{y}^{2}$​ the variances, and Cov(x,y) the covariance between the two feature sets (e.g., non‐PVC and PVC‐corrected). CCC interpretation:

- Almost perfect agreement: ≥ 0.99
- Substantial agreement: 0.95–0.99
- Moderate agreement: 0.80–0.95
- Poor agreement: < 0.80

To visualize these patterns, heatmaps and boxplots were used to highlight reproducibility differences across feature classes and organs.

### 2.6 Qualitative visual assessment

To evaluate the clinical image quality of different PVC methods, a blinded visual assessment was conducted independently by two board‐certified nuclear medicine specialists. Each reader independently scored reconstructed SPECT images corresponding to three processing methods: uncorrected SPECT (No PVC), RL‐based PVC, and RVC‐based PVC. The readers were blinded to the reconstruction type and the image presentation order was randomized. Eight key qualitative criteria were evaluated for each image: Image Quality, Lesion Contrast, Noise, Edge Definition / Sharpness, Diagnostic Confidence, Background Homogeneity, Anatomical Accuracy, and Overall Preference. A 6‐point Likert scale was used for scoring: 0 = Non diagnostic, 1 = Poor, 2 = Satisfactory, 3 = Good, 4 = Very Good, and 5 = Excellent. All evaluations were performed using standardized color scales and windowing presets. The readers were instructed to assess each image independently without discussing their scores with one another. This setup enabled both within‐reader analysis and inter‐reader agreement evaluation.

### 2.7 Statistical analysis

All statistical analyses were performed using Python (version 3.1). Since visual image scores were ordinal and based on repeated evaluations, non‐parametric tests were employed to assess differences across reconstruction methods. For each reader, the Friedman test was used to assess global differences among the three PVC strategies, uncorrected SPECT (No PVC), RL‐based PVC, and RVC‐based PVC, for each qualitative scoring criterion. Post hoc comparisons were conducted using the Wilcoxon signed‐rank test to evaluate pairwise differences. To assess inter‐reader agreement, two complementary methods were employed: (1) Spearman rank correlation to evaluate monotonic consistency across readers; and (2) Cohen's weighted Kappa with quadratic weights to quantify ordinal agreement for each criterion. A *p*‐value of < 0.05 was considered statistically significant for all hypothesis tests.

### 2.8 Tumor burden indices

To provide an exploratory volumetric description of hepatic metastatic tumor burden in the theranostic setting, we employed two indices: the MLR and metastasis‐to‐spare‐liver ratio (MSLR), defined as:

- MLR = MeshVolume (Metastasis) / MeshVolume (Liver)
- MSLR = MeshVolume (Metastasis) / MeshVolume (Liver − Metastasis)

MLR reflects the relative metastatic tumor volume within the entire liver, whereas MSLR normalizes tumor burden to the spared (non‐tumorous) liver parenchyma. Volumes were derived using the MeshVolume feature from the 3D shape category based on liver and metastasis segmentations in post‐therapy ^1^ ^7^ ^7^Lu SPECT/CT images.

## 3 RESULTS

Table 1 summarizes visual scoring results from two board‐certified nuclear medicine physicians. Global differences across the three reconstruction methods (No PVC, RL, RVC) were assessed using the Friedman test, followed by post hoc Wilcoxon signed‐rank tests (*p* < 0.05) for pairwise comparisons. RL yielded the highest scores in 5 of 8 criteria for Reader 1, with significant improvements in Image Quality (*χ* ^2^ = 9.48, *p* = 0.0087), Lesion Contrast (*χ* ^2^ = 13.15, *p* = 0.0014), and Sharpness (*χ* ^2^ = 14.60, *p* = 0.0007), e.g., Image Quality: RL = 3.25 versus SPECT = 2.93 (*p* = 0.009). Reader 2 reported stronger differences, especially for Lesion Contrast (*χ* ^2^ = 35.20), Noise (*χ* ^2^ = 45.76), and Background Homogeneity (*χ* ^2^ = 50.08), all *p* < 0.0001, with both RL and RVC significantly outperforming uncorrected SPECT (e.g., Noise: RL = 2.48 vs. SPECT = 3.25, *p* < 0.0001). Inter‐reader variability was observed, with Reader 1 favoring RL and Reader 2 recognizing strengths in both PVC strategies.

**TABLE 1:** Blinded visual scoring results for three reconstruction methods (No PVC, RL, RVC), evaluated independently by two nuclear medicine specialists using eight 6‐point Likert criteria. Friedman and Wilcoxon tests were used to assess global and pairwise differences. Mean scores and *p*‐values are reported; RL versus RVC for anatomical accuracy was not computed due to identical ratings.

Criterion	Friedman Chi‐Square	*p*‐value	Wilcoxon test: SPECT vs. RL	Wilcoxon test: SPECT vs. RVC	Wilcoxon test: RL vs. RVC	SPECT mean per method	RL mean per method	RVC mean per method
Image quality								
Physician 1	9.48	0.01	0.01	0.12	0.10	2.92	3.25	3.10
Physician 2	5.78	0.06	0.23	0.11	0.18	3.20	3.33	3.40
Lesion contrast								
Physician 1	13.15	0.00	0.00	0.02	0.13	2.90	3.30	3.15
Physician 2	35.20	0.00	0.00	0.00	0.18	3.18	3.70	3.78
Noise								
Physician 1	0.66	0.72	0.32	1.00	0.56	3.45	3.42	3.45
Physician 2	45.76	0.00	0.00	0.00	0.32	3.25	2.48	2.45
Edge definition / sharpness								
Physician 1	14.60	0.00	0.00	0.04	0.12	2.90	3.32	3.15
Physician 2	27.44	0.00	0.00	0.00	0.32	3.00	3.48	3.53
Diagnostic confidence								
Physician 1	2.96	0.23	0.18	0.73	0.10	2.72	2.80	2.70
Physician 2	0.29	0.87	0.71	1.00	0.32	3.68	3.65	3.68
Background homogeneity								
Physician 1	0.92	0.63	0.71	0.36	0.31	3.50	3.47	3.42
Physician 2	50.07	0.00	0.00	0.00	0.32	3.55	2.85	2.90
Anatomical Accuracy								
Physician 1	0.00	1.00	1.00	1.00	1.00	2.50	2.50	2.50
Physician 2	2.00	0.37	0.32	0.32	Nan	3.30	3.28	3.28
Overall preference								
Physician 1	9.32	0.01	0.01	0.06	0.27	2.22	1.75	1.95
Physician 2	7.02	0.03	0.03	0.07	1.00	2.43	2.18	2.18

Table S1 summarizes ICC‐based reproducibility of 837 features across 11 regions at BW50; results for other bin widths are in Tables S2–S4. Original features showed the highest ICCs (e.g., liver: 98.9%), while high‐frequency wavelet features were more variable, especially in small or complex organs. Lower bin widths yielded more stable features, with ICC dropping by 20%–30% at BW200.

CCC‐based analysis showed that RL–RVC pairs consistently achieved the highest agreement, particularly at lower bin widths (BW50–100), with over 85% of features in organs like the liver, pancreas, and IVC reaching CCC ≥ 0.90. As bin width increased to BW150–200, agreement declined by 20%–30%, especially for small or complex organs (e.g., kidneys, stomach), where > 40% of features fell below CCC of 0.80. RL–RVC agreement remained stronger than comparisons involving uncorrected SPECT. Full organ‐wise distributions are provided in Table S5.

Inter‐rater CCC analysis showed that approximately one‐third of features achieved Substantial or Almost Perfect agreement at BW50–100, whereas the majority remained in the Poor‐to‐Moderate range, highlighting segmentation variability as an important source of feature instability (Table 2). The principal reproducibility trends across PVC strategies and discretization settings are summarized in Table 3, providing a compact overview of the most robust feature categories and organ regions.

**TABLE 2:** Distribution of radiomics features across qualitative CCC agreement categories (poor, moderate, substantial, almost perfect) for inter‐rater reproducibility. Segmentations were independently performed by two nuclear medicine specialists, and CCC was computed at four discretization bin widths (50, 100, 150, 200). Values represent the number of features (out of 837) in each category. Overall, roughly one‐third of features achieved substantial or almost perfect agreement, whereas the remainder were classified as moderate or poor.

	Poor	Moderate	Substantial	Almost perfect
SPECT‐BIN50	185	382	188	82
SPECT‐BIN100	164	381	208	84
SPECT‐BIN150	169	366	205	97
SPECT‐BIN200	167	364	218	88
RL‐BIN50	218	369	166	84
RL‐BIN100	203	362	189	83
RL‐BIN150	189	383	190	75
RL‐BIN200	183	371	204	79
RVC‐BIN50	215	364	177	81
RVC‐BIN100	184	379	198	76
RVC‐BIN150	168	390	196	83
RVC‐BIN200	187	360	210	80

**TABLE 3:** A comprehensive comparison of PVC methods in theranostic ^177^Lu SPECT/CT radiomics. This table evaluates the three reconstruction strategies—Richardson–Lucy (RL), Reblurred Van Cittert (RVC), and uncorrected SPECT—Across multiple performance dimensions, including feature reproducibility (ICC/CCC), visual quality improvement, anatomical consistency, and theranostics applicability.

PVC method	Reproducibility (ICC/CCC)	Stable features (ICC ≥ 0.9)	Unstable features (ICC < 0.5)	Visual score improvement	Organ sensitivity	Consistency across bin widths	CCC agreement with other methods	Robustness in original SPECT (PVC‐dependence)	Theranostics utility	Modeling recommendation	Clinical utility	Key notes	Final recommendation
RL (Richardson–Lucy)	High ICC for Original and LLL‐wavelet; Strong CCC with RVC (≥0.95 at BW50–100)	Highest proportion, mostly in large/homogeneous organs	Few; primarily high‐frequency wavelet features	+0.3–0.4 Likert points (Reader 1); consistent but flatter for Reader 2	Stable in liver, IVC, spleen	High	CCC > 0.95 with RVC	Moderate dependency	High — reliable	Ideal for sensitive modeling	Oncology, liver metastasis response monitoring, personalized treatment	Most reproducible; strong visual and radiomics performance	Highly recommended; consistent across metrics and readers
RVC (Reblurred Van Cittert)	Moderate‐to‐high ICC; Good CCC with RL (≥0.90)	High, but slightly lower than RL, especially for small organs	Low‐to‐moderate; mainly high‐frequency features	+0.2–0.3 Likert points; no strong preference over RL (Reader 2)	Stable in liver, pancreas; more variable in stomach	Moderate	CCC > 0.95 with RL	High PVC‐dependence	Moderate – high	Suitable for exploratory analyses	Adjunctive modeling, therapy response estimation	Close to RL; slightly lower ICC in wavelet sets	Recommended; suitable alternative when RL not feasible
Uncorrected SPECT (No PVC)	Low‐to‐moderate ICC; Lower CCC (< 0.85)	Moderate; mostly original features in large organs	Highest; sensitive to bin width and wavelet frequency	Baseline	Poor reproducibility in kidneys, pancreas	Low	Weak — CCC < 0.85 with PVCs	Low baseline performance	Limited — inconsistent	Use with caution; select stable features	Visual screening only, pre‐processing phase	High instability, low ICC; limited for modeling. Both readers rated it lowest across most criteria.	Not recommended

**FIGURE 3:**
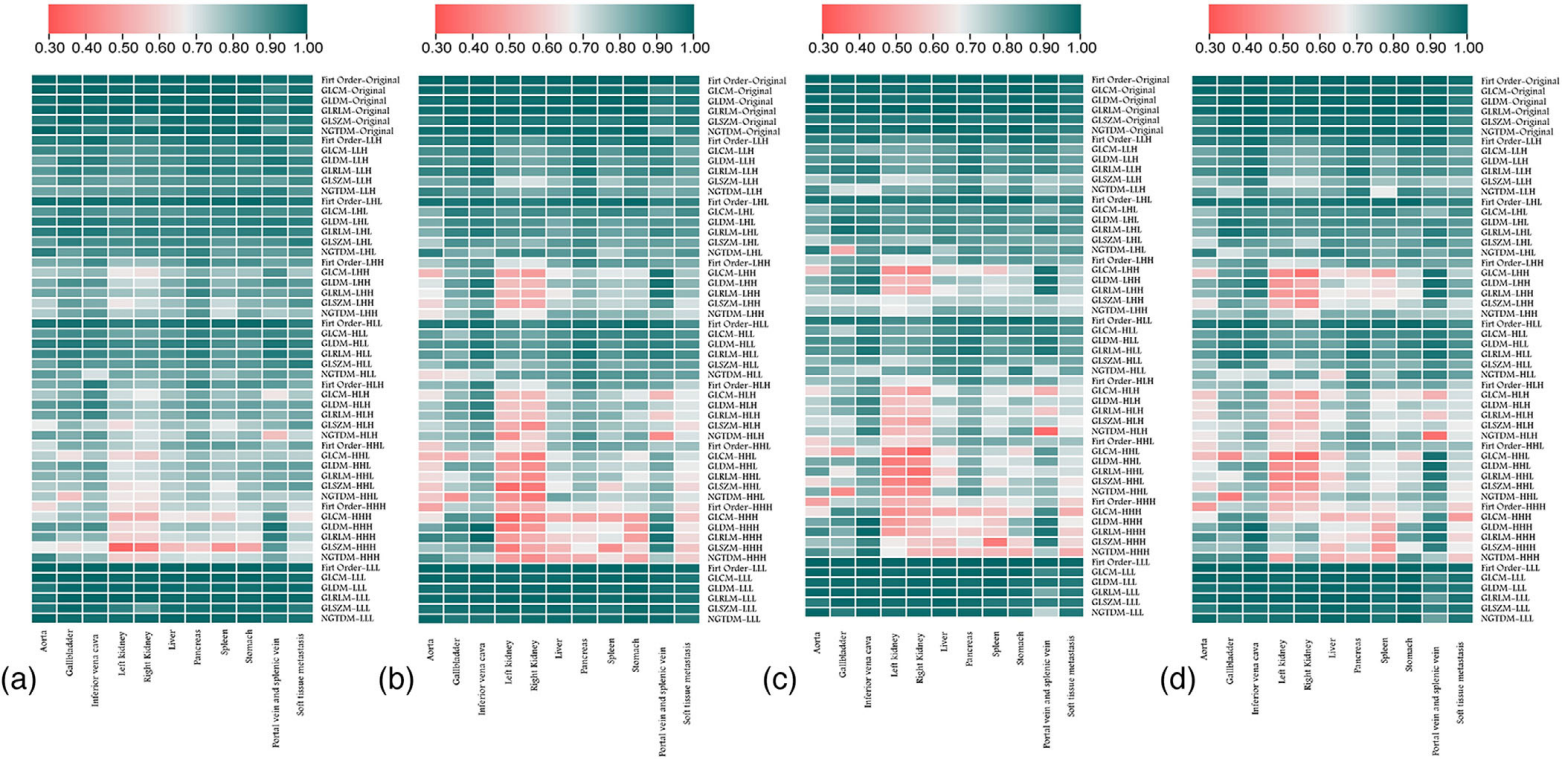
Heatmaps of ICC‐based radiomics feature reproducibility across organs and feature groups for different bin widths. This figure presents heatmaps illustrating the reproducibility of radiomics features across 11 anatomical regions and soft tissue metastasis (rows) and 9 feature groups (columns) based on Intraclass Correlation Coefficient (ICC) scores. Each heatmap corresponds to a different bin width used during intensity discretization in feature extraction: (A) Bin width = 50, (B) Bin width = 100, (C) Bin width = 150, and (D) Bin width = 200. The vertical axis lists the evaluated anatomical structures: aorta, gallbladder, inferior vena cava, left kidney, right kidney, liver, pancreas, spleen, stomach, portal vein and splenic vein, and soft tissue metastases. The horizontal axis includes the original feature set and eight wavelet‐transformed feature groups (LLH, LHL, LHH, HLL, HLH, HHL, HHH, LLL). All panels are displayed using the same ICC color scale to enable direct comparison across bin widths.

Figure 3 shows ICC heatmaps of 837 radiomic features across 11 regions and four bin widths (BW50–BW200), grouped by feature class. Reproducibility declined with increasing bin width. At BW50, most original and low‐frequency wavelet features (LLH, LLL) achieved ICC ≥ 0.90 in stable organs like the liver and IVC. In contrast, high‐frequency decompositions (e.g., HLH, HHH, HHL) exhibited greater instability at BW200, especially in complex organs, such as the pancreas, spleen, and kidneys (e.g., Right Kidney GLDM_HHH: ICC ∼0.40). Original features remained consistently robust, while high‐frequency wavelets frequently dropped below ICC of 0.60 at coarser discretization.

Statistical significance analysis (*p* < 0.05) of ICC‐based reproducibility demonstrated that the proportion of robust radiomic features remained consistently high across all abdominal organs, with a mild decrease observed at coarser discretization settings. Specifically, 98.9% of features were statistically significant at BW50, declining slightly to 95.8% at BW100, 93.6% at BW150, and 90.1% at BW200.

**FIGURE 4:**
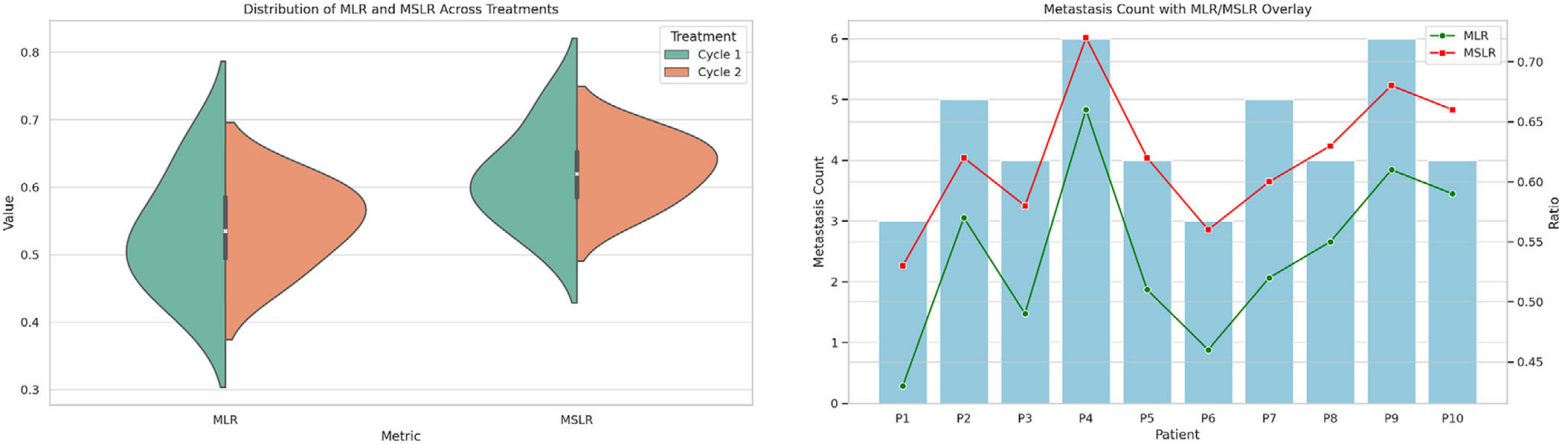
Exploratory visualization of metastatic liver tumor burden using MLR and MSLR indices. Violin plots summarize the distribution of MLR/MSLR values across patients, and the metastasis‐count overlay illustrates their relationship with lesion burden. Due to incomplete paired imaging across treatment cycles, the results are descriptive and do not represent within‐patient longitudinal changes.

Figure 4 presents exploratory representations of hepatic metastatic tumor burden using the MLR and metastasis‐to‐spare‐liver ratio (MSLR) derived from post‐therapy ^1^ ^7^ ^7^Lu SPECT/CT. Because paired longitudinal imaging across both treatment cycles was not available for most patients, these metrics were analyzed descriptively rather than as within‐patient response measures. MLR values ranged from 0.43 to 0.66 and MSLR values from 0.53 to 0.72, indicating substantial inter‐patient variability. The violin plots summarize the overall distribution of these indices, while the metastasis‐count overlay illustrates their relationship with lesion burden. These preliminary findings suggest that MLR/MSLR may serve as volumetric markers of metastatic liver involvement, warranting validation in larger longitudinal cohorts.

**FIGURE 5:**
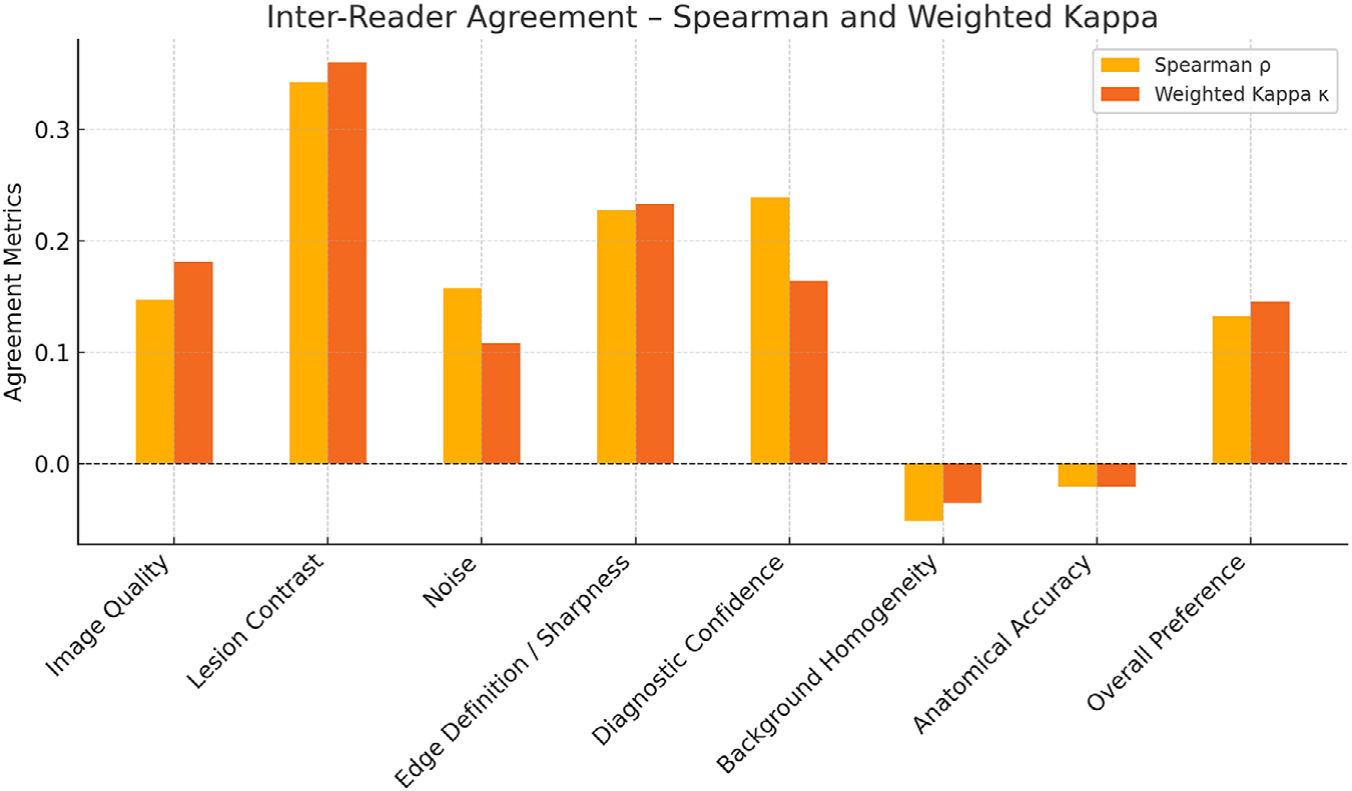
Inter‐reader agreement between the two nuclear medicine physicians across eight qualitative criteria. Agreement was quantified using Spearman's rank correlation coefficient (*ρ*, yellow) to measure consistency in relative ranking of scores, and Cohen's weighted Kappa (*κ*, orange) to measure agreement on the ordinal 6‐point Likert scale. Values close to 1 indicate strong agreement, whereas values near 0 or negative indicate poor concordance.

Figure 5 shows inter‐reader agreement across eight qualitative criteria using Spearman's *ρ* and weighted Kappa (*κ*). The highest concordance was found for Lesion Contrast (*ρ* = 0.35, *κ* = 0.37) and Edge Definition/Sharpness (*ρ* = 0.23, *κ* = 0.23), reflecting modest consistency. In contrast, Background Homogeneity and Anatomical Accuracy showed near‐zero or negative values (*ρ*, *κ* < 0), indicating substantial disagreement in reader interpretations.

## 4 DISCUSSION

Radiomics analysis enables quantitative characterization of disease from SPECT/CT imaging, offering potential for personalized theranostic strategies. However, its clinical translation is limited by poor reproducibility of features across reconstruction settings and correction methods.^53^, ^54^ In theranostic ^177^Lu imaging, these challenges are amplified by noise, resolution limits, and PVE.^24^, ^55^ Implementing standardized and robust PVC techniques is therefore essential to ensure feature stability and support reliable treatment response monitoring.^56^ Although PVC does not inherently guarantee improved predictive performance, identifying radiomics features that remain reproducible across commonly used correction strategies is a necessary methodological prerequisite for downstream radiomics modeling and future clinical translation in theranostic SPECT.

This study provides a systematic single‐center evaluation of radiomics feature reproducibility in theranostic ^1^ ^7^ ^7^Lu SPECT/CT, focusing on abdominal organs and liver metastases. Using ICC and CCC, we assessed the stability of non‐shape features across different PVC methods (RL, RVC) and discretization settings, identifying robust versus variable feature‐organ combinations. In addition to reproducibility, we included qualitative image assessments and exploratory use of volumetric biomarkers (MLR, MSLR) for evaluating metastatic liver burden. While RL showed improved agreement and visual quality, its utility for treatment response monitoring remains exploratory and warrants further validation.

As summarized in Table 3, the principal findings highlight the impact of PVC strategies on radiomics feature reproducibility in ^1^ ^7^ ^7^Lu SPECT/CT. Unlike previous work in brain PET,^22^ which primarily emphasized COV stability and cortical mapping, the present study focused on abdominal oncological imaging, integrating reproducibility metrics with qualitative image assessment and exploratory tumor burden indices relevant to the theranostic treatment setting.

Among the evaluated methods, RL emerged as the most consistent in terms of overall agreement and perceived image quality, without demonstrating uniformly excellent reproducibility across all radiomics features. It showed high reproducibility for several original and LLL‐wavelet features at lower bin widths and achieved strong agreement with RVC (CCC > 0.95 across most organs at BW50–100). Visual scoring by nuclear medicine specialists indicated a modest improvement in sharpness and contrast (mean Likert +0.3–0.4). Although PVC improved perceived image quality, the present study does not propose PVC‐corrected images for routine diagnostic interpretation, as this remains task‐dependent and requires dedicated clinical validation. Feature stability was highest in large, homogeneous organs (e.g., liver, spleen, IVC). RVC showed more variability in wavelet features and smaller organs but maintained good agreement with RL in key regions. RL appears preferable for pipelines requiring both stability and interpretable image quality, based mainly on agreement and visual scoring. Inter‐rater reproducibility analysis revealed that many features showed only poor‐to‐moderate CCC, underscoring segmentation variability as a critical limitation. These findings highlight the importance of standardized or consensus‐based delineation protocols to reduce operator dependence.

SPECT without PVC demonstrated the lowest reproducibility and agreement, with ICC values often < 0.75 for high‐frequency features, increased variability at coarser bin widths, and CCC < 0.85 in most pairwise comparisons. While uncorrected SPECT remains adequate for qualitative assessments, its limitations for high‐precision radiomics and theranostics modeling are evident. Shape‐based volumetric biomarkers (MLR and MSLR) were explored descriptively as indices of metastatic liver burden, although paired longitudinal assessment was not available for most patients. Because these indices are derived from identical segmentation masks, they primarily capture disease progression rather than differences between PVC methods, although RL‐based reconstructions facilitated more discernible volumetric shifts for clinical interpretation and adaptive planning.

From a modeling perspective, the study also provided clear guidance: RL may provide a more stable feature space that could support future response modeling studies, pending prospective validation. Both methods demonstrated strong utility in feature classes like GLCM, GLDM, and First‐order, which retained stability across organs and discretization schemes. Conversely, high‐frequency wavelet groups (HLH, HHL, HHH) and anatomically complex organs (kidneys, pancreas) were more prone to variability, suggesting the need for region‐aware and feature‐aware filtering in downstream applications.

Inter‐reader agreement analysis (Figure 5) showed only modest concordance across visual criteria, with particularly low agreement for Anatomical Accuracy and Background Homogeneity. These discrepancies mainly reflected differing preferences between RL and RVC rather than doubts about the overall benefit of PVC. Reader 2 favored both PVC methods over uncorrected SPECT, while Reader 1 showed a clear preference for RL. This supports the general perception that PVC enhances image quality, despite inter‐reader variability. These results underscore the need for future studies with more observers and standardized evaluation frameworks to better assess the clinical value of PVC.

Despite the promising findings, several limitations should be acknowledged. First, this study was conducted in a single‐center setting and involved a relatively small patient cohort (*n* = 13 patients, 40 post‐therapy scans), which may limit the generalizability of the results across imaging systems, protocols, and clinical populations. In addition, the inclusion of multiple post‐therapy scans from the same individuals may introduce patient‐specific correlations, further underscoring the need for larger independent cohorts. Multi‐institutional validation will be essential to confirm reproducibility across broader contexts. Therefore, the findings should be interpreted as preliminary reproducibility trends within a controlled clinical acquisition environment. Broader multi‐center harmonization studies will be required before clinical generalization. Second, although this study incorporated qualitative assessments from two independent nuclear medicine specialists in a blinded setting, the number of readers remains limited. Notably, the inter‐reader agreement was modest (*κ* < 0.4 for most criteria), with perceptual differences evident in scoring individual PVC strategies. While both readers consistently rated PVC‐corrected images higher than uncorrected SPECT, their qualitative scores differed regarding whether RL or RVC provided greater perceived improvement, highlighting subjective variability in visual assessment. These findings underscore the inherent subjectivity of visual scoring and reinforce the need for broader multi‐reader validation in future studies to enhance reproducibility and reduce interpretation bias.

Third, only a single SPECT reconstruction protocol was used in this study. Given the known influence of reconstruction parameters on radiomics features, future studies should investigate reproducibility across different vendors, filter settings, and reconstruction algorithms to ensure broader applicability. Because RR‐based OSEM reconstruction produces spatially varying resolution across the FOV, the assumption of a stationary PSF in post‐reconstruction PVC may not fully hold, representing an additional limitation of RL/RVC correction in clinical ^1^ ^7^ ^7^Lu SPECT/CT. Fourth, MLR and MSLR were explored descriptively as volumetric indices of metastatic liver burden; however, incomplete paired imaging across treatment cycles limited interpretation as response measures. In some cases, rising MLR/MSLR values were not matched by increased metastasis counts, likely due to liver volume changes, segmentation variability, mixed treatment response, and differences in image quality. These findings suggest that relative indices like MLR/MSLR should be interpreted alongside absolute lesion uptake for a more accurate evaluation of therapy response in SPECT/CT radiomics.

This study focused on two post‐reconstruction PVC methods, RL and RVC, chosen for their clinical availability, compatibility with open‐source tools (e.g., PETPVC), and prior validation in SPECT imaging.^17^, ^18^ While their simplicity supports reproducibility and integration into clinical workflows, this limited the scope of PVC evaluation. Future studies should explore more advanced methods, including region‐based, voxel‐wise, or deep learning‐based PVC,^57^ particularly in anatomically complex or noisy theranostic ^1^ ^7^ ^7^Lu settings.

Building on these findings, future efforts should prioritize multi‐center validation across diverse scanners and protocols to ensure generalizability. Outcome‐based modeling linking robust radiomics features and volumetric indices (e.g., MLR, MSLR) to clinical endpoints is needed. Delta‐radiomics, shape‐based metrics, and temporal changes may further enhance biological interpretation. Technically, incorporating deep learning‐based PVC and automated segmentation can reduce variability. Establishing reproducibility benchmarks that integrate texture, dosimetry, and uptake metrics will support personalized ^1^ ^7^ ^7^Lu SPECT/CT theranostics.

## 5 CONCLUSION

In this study, we assessed radiomics feature reproducibility in ^1^ ^7^ ^7^Lu SPECT/CT using two post‐reconstruction PVC strategies (RL and RVC) versus uncorrected images. Reproducibility was highest in large, homogeneous organs (e.g., liver, IVC) and for original or low‐frequency wavelet features, while high‐frequency features and complex organs (e.g., kidneys, pancreas) were more variable. RL and RVC showed stronger mutual agreement than either did with uncorrected SPECT, suggesting that reproducibility depends more on feature class and organ than on PVC choice alone. Some texture features, such as GLCM Correlation and GLDM Dependence NonUniformity, demonstrated high reproducibility even without PVC. Additionally, volumetric indices (MLR, MSLR) increased across therapy cycles, reflecting tumor burden dynamics rather than PVC effects. Segmentation differences also impacted feature stability, reinforcing the importance of consistent VOI delineation. Overall, these findings emphasize that reproducibility in SPECT radiomics is feature‐ and organ‐dependent, and is further influenced by scanner‐specific characteristics and reconstruction protocols. Methodological standardization of reconstruction, discretization parameters, and PVC strategies is an important prerequisite for improving the robustness of SPECT radiomics workflows and for supporting future studies aiming at clinical translation of quantitative imaging biomarkers.

## FUNDING INFORMATION

This work was supported by the Swiss National Science Foundation under grant SNSF 30030–231742 by Geneva League Against Cancer under grant LGC 2402.

## CONFLICT OF INTEREST STATEMENT

The authors declare that they have no conflicts of interest.

## ETHICAL APPROVAL

The study protocol was approved by the Institutional Review Board of xxx (IRB #RAD0343). All patient data were anonymized prior to analysis. Given the retrospective design and anonymized datasets, the requirement for written informed consent was waived.

## Supporting information



Supporting Information

## Data Availability

The imaging data used in this study are not publicly available owing to ethical and institutional restrictions. The code developed for the analysis is available upon request from the corresponding author.
