# Supplementary material for: Impact of partial volume correction on radiomics reproducibility in theranostic SPECT/CT imaging
Source: Med Phys. 2026 Apr 3;53(4):e70427. doi: 10.1002/mp.70427 (PMC13048878; doi:10.1002/mp.70427)
Supplement: Supplementary file 1 — Supporting Information [file MP-53-0-s001.pdf]

Supplementary Material

Table S1. Distribution reproducibility scores (ICC-based: Excellent, Good, Moderate, Poor) for 837 radiomic features across 11 organs (aorta, gallbladder, inferior vena cava (IVC), left kidney, right kidney, liver, pancreas, spleen, stomach, portal vein and splenic vein, and soft tissue metastases) and 9 feature groups (Original and eight wavelet-transformed groups) using a bin width of 50.

	Quality	STM	PSV	Stomach	Spleen	Pancreas	Liver	RK	LK	IVC	GB	Aorta
**Original**	Excellent	89 (96%)	72 (77%)	89 (96%)	92 (99%)	92 (99%)	92 (99%)	87 (94%)	90 (97%)	87 (94%)	92 (99%)	90 (97%)
	Good	4 (4%)	14 (15%)	4 (4%)	1 (1%)	1 (1%)	1 (1%)	3 (3%)	3 (3%)	5 (5%)	0 (0%)	2 (2%)
	Moderate	0 (0%)	6 (6%)	0 (0%)	0 (0%)	0 (0%)	0 (0%)	3 (3%)	0 (0%)	1 (1%)	0 (0%)	0 (0%)
	Poor	0 (0%)	1 (1%)	0 (0%)	0 (0%)	0 (0%)	0 (0%)	0 (0%)	0 (0%)	0 (0%)	1 (1%)	1 (1%)
**LLH**	Excellent	59 (63%)	70 (75%)	68 (73%)	65 (70%)	76 (82%)	64 (69%)	44 (47%)	45 (48%)	71 (76%)	65 (70%)	63 (68%)
	Good	28 (30%)	20 (22%)	24 (26%)	26 (28%)	16 (17%)	25 (27%)	44 (47%)	37 (40%)	15 (16%)	23 (25%)	22 (24%)
	Moderate	6 (6%)	2 (2%)	1 (1%)	2 (2%)	0 (0%)	4 (4%)	5 (5%)	10 (11%)	7 (8%)	4 (4%)	5 (5%)
	Poor	0 (0%)	1 (1%)	0 (0%)	0 (0%)	1 (1%)	0 (0%)	0 (0%)	1 (1%)	0 (0%)	1 (1%)	3 (3%)
**LHL**	Excellent	60 (65%)	66 (71%)	72 (77%)	68 (73%)	72 (77%)	65 (70%)	63 (68%)	63 (68%)	69 (74%)	77 (83%)	66 (71%)
	Good	31 (33%)	23 (25%)	18 (19%)	19 (20%)	20 (22%)	24 (26%)	27 (29%)	26 (28%)	22 (24%)	13 (14%)	18 (19%)
	Moderate	2 (2%)	3 (3%)	3 (3%)	6 (6%)	1 (1%)	4 (4%)	3 (3%)	2 (2%)	2 (2%)	2 (2%)	9 (10%)
	Poor	0 (0%)	1 (1%)	0 (0%)	0 (0%)	0 (0%)	0 (0%)	0 (0%)	2 (2%)	0 (0%)	1 (1%)	0 (0%)
**LHH**	Excellent	44 (47%)	47 (51%)	24 (26%)	21 (23%)	50 (54%)	26 (28%)	18 (19%)	15 (16%)	47 (51%)	34 (37%)	24 (26%)
	Good	26 (28%)	27 (29%)	49 (53%)	44 (47%)	35 (38%)	39 (42%)	17 (18%)	25 (27%)	29 (31%)	37 (40%)	34 (37%)
	Moderate	21 (23%)	18 (19%)	20 (22%)	24 (26%)	8 (9%)	26 (28%)	52 (56%)	46 (49%)	10 (11%)	20 (22%)	33 (35%)
	Poor	2 (2%)	1 (1%)	0 (0%)	4 (4%)	0 (0%)	2 (2%)	6 (6%)	7 (8%)	7 (8%)	2 (2%)	2 (2%)
**HLL**	Excellent	60 (65%)	72 (77%)	68 (73%)	63 (68%)	75 (81%)	57 (61%)	45 (48%)	46 (49%)	64 (69%)	67 (72%)	55 (59%)
	Good	31 (33%)	19 (20%)	16 (17%)	19 (20%)	16 (17%)	28 (30%)	36 (39%)	39 (42%)	25 (27%)	21 (23%)	35 (38%)
	Moderate	2 (2%)	1 (1%)	9 (10%)	8 (9%)	2 (2%)	7 (8%)	10 (11%)	6 (6%)	3 (3%)	4 (4%)	2 (2%)
	Poor	0 (0%)	1 (1%)	0 (0%)	3 (3%)	0 (0%)	1 (1%)	2 (2%)	2 (2%)	1 (1%)	1 (1%)	1 (1%)
**HLH**	Excellent	38 (41%)	28 (30%)	25 (27%)	18 (19%)	36 (39%)	25 (27%)	18 (19%)	17 (18%)	57 (61%)	34 (37%)	20 (22%)
	Good	21 (23%)	23 (25%)	44 (47%)	47 (51%)	45 (48%)	37 (40%)	28 (30%)	25 (27%)	24 (26%)	32 (34%)	40 (43%)
	Moderate	31 (33%)	36 (39%)	23 (25%)	22 (24%)	12 (13%)	30 (32%)	39 (42%)	44 (47%)	11 (12%)	25 (27%)	30 (32%)
	Poor	3 (3%)	6 (6%)	1 (1%)	6 (6%)	0 (0%)	1 (1%)	8 (9%)	7 (8%)	1 (1%)	2 (2%)	3 (3%)
**HHL**	Excellent	38 (41%)	33 (35%)	13 (14%)	18 (19%)	21 (23%)	18 (19%)	13 (14%)	13 (14%)	32 (34%)	22 (24%)	15 (16%)
	Good	25 (27%)	30 (32%)	50 (54%)	36 (39%)	45 (48%)	37 (40%)	19 (20%)	28 (30%)	31 (33%)	32 (34%)	36 (39%)
	Moderate	28 (30%)	26 (28%)	28 (30%)	30 (32%)	25 (27%)	31 (33%)	41 (44%)	28 (30%)	28 (30%)	28 (30%)	36 (39%)
	Poor	2 (2%)	4 (4%)	2 (2%)	9 (10%)	2 (2%)	7 (8%)	20 (22%)	24 (26%)	2 (2%)	11 (12%)	6 (6%)
**HHH**	Excellent	19 (20%)	51 (55%)	12 (13%)	10 (11%)	19 (20%)	10 (11%)	8 (9%)	10 (11%)	34 (37%)	27 (29%)	29 (31%)
	Good	22 (24%)	33 (35%)	25 (27%)	11 (12%)	24 (26%)	24 (26%)	10 (11%)	9 (10%)	29 (31%)	26 (28%)	27 (29%)
	Moderate	31 (33%)	8 (9%)	34 (37%)	49 (53%)	29 (31%)	45 (48%)	29 (31%)	22 (24%)	21 (23%)	32 (34%)	22 (24%)
	Poor	21 (23%)	1 (1%)	22 (24%)	23 (25%)	21 (23%)	14 (15%)	46 (49%)	52 (56%)	9 (10%)	8 (9%)	15 (16%)

**LLL**	Excellent	89 (96%)	88 (95%)	87 (94%)	90 (97%)	87 (94%)	93 (100%)	88 (95%)	87 (94%)	90 (97%)	91 (98%)	85 (91%)
	Good	4 (4%)	3 (3%)	6 (6%)	3 (3%)	6 (6%)	0 (0%)	2 (2%)	3 (3%)	2 (2%)	0 (0%)	4 (4%)
	Moderate	0 (0%)	0 (0%)	0 (0%)	0 (0%)	0 (0%)	0 (0%)	0 (0%)	2 (2%)	1 (1%)	0 (0%)	4 (4%)
	Poor	0 (0%)	2 (2%)	0 (0%)	0 (0%)	0 (0%)	0 (0%)	3 (3%)	1 (1%)	0 (0%)	2 (2%)	0 (0%)

Table S2. Distribution of radiomics feature reproducibility scores (ICC-based: Excellent, Good, Moderate, Poor) across 11 organs (aorta, gallbladder, inferior vena cava (IVC), left kidney, right kidney, liver, pancreas, spleen, stomach, portal vein and splenic vein, and soft tissue metastases) and 9 feature groups (Original and eight wavelet-transformed groups) using a bin width of 100.

	Quality	STM	PSV	Stomach	Spleen	Pancreas	Liver	RK	LK	IVC	GB	Aorta
**Original**	Excellent	88 (95%)	73 (78%)	92 (99%)	89 (96%)	88 (95%)	87 (94%)	88 (95%)	88 (95%)	93 (100%)	90 (97%)	89 (96%)
	Good	5 (5%)	9 (10%)	1 (1%)	3 (3%)	4 (4%)	6 (6%)	5 (5%)	4 (4%)	0 (0%)	2 (2%)	3 (3%)
	Moderate	0 (0%)	9 (10%)	0 (0%)	1 (1%)	1 (1%)	0 (0%)	0 (0%)	1 (1%)	0 (0%)	0 (0%)	0 (0%)
	Poor	0 (0%)	1 (2%)	0 (0%)	0 (0%)	0 (0%)	0 (0%)	0 (0%)	0 (0%)	0 (0%)	1 (1%)	1 (1%)
**LLH**	Excellent	34 (37%)	65 (70%)	63 (68%)	43 (46%)	84 (90%)	49 (53%)	22 (24%)	28 (30%)	81 (87%)	62 (67%)	58 (62%)
	Good	48 (52%)	21 (23%)	26 (28%)	41 (44%)	8 (9%)	32 (34%)	52 (56%)	49 (53%)	11 (12%)	26 (28%)	30 (32%)
	Moderate	11 (12%)	4 (4%)	4 (4%)	7 (8%)	0 (0%)	10 (11%)	19 (20%)	13 (14%)	1 (1%)	2 (2%)	4 (4%)
	Poor	0 (0%)	3 (3%)	0 (0%)	2 (2%)	1 (1%)	2 (2%)	0 (0%)	3 (3%)	0 (0%)	3 (3%)	1 (1%)
**LHL**	Excellent	50 (54%)	44 (47%)	60 (65%)	66 (71%)	67 (72%)	58 (62%)	51 (55%)	47 (51%)	68 (73%)	67 (72%)	45 (48%)
	Good	37 (40%)	42 (45%)	22 (24%)	15 (16%)	24 (26%)	24 (26%)	35 (38%)	38 (41%)	20 (22%)	20 (22%)	29 (31%)
	Moderate	6 (6%)	5 (5%)	10 (11%)	8 (9%)	1 (1%)	7 (8%)	7 (8%)	7 (8%)	4 (4%)	4 (4%)	16 (17%)
	Poor	0 (0%)	2 (2%)	1 (1%)	4 (4%)	1 (1%)	4 (4%)	0 (0%)	1 (1%)	1 (1%)	2 (2%)	3 (3%)
**LHH**	Excellent	24 (26%)	69 (74%)	17 (18%)	17 (18%)	27 (29%)	23 (25%)	9 (10%)	11 (12%)	62 (67%)	37 (40%)	12 (13%)
	Good	30 (32%)	18 (19%)	34 (37%)	40 (43%)	40 (43%)	28 (30%)	7 (8%)	8 (9%)	22 (24%)	26 (28%)	16 (17%)
	Moderate	36 (39%)	5 (5%)	39 (42%)	27 (29%)	25 (27%)	29 (31%)	32 (34%)	32 (34%)	9 (10%)	27 (29%)	43 (46%)
	Poor	3 (3%)	1 (1%)	3 (3%)	9 (10%)	1 (1%)	13 (14%)	45 (48%)	42 (45%)	0 (0%)	3 (3%)	22 (24%)
**HLL**	Excellent	43 (46%)	74 (80%)	71 (76%)	60 (65%)	79 (85%)	58 (62%)	38 (41%)	36 (39%)	65 (70%)	46 (49%)	44 (47%)
	Good	46 (49%)	15 (16%)	17 (18%)	23 (25%)	14 (15%)	27 (29%)	37 (40%)	33 (35%)	26 (28%)	30 (32%)	38 (41%)
	Moderate	4 (4%)	3 (3%)	4 (4%)	8 (9%)	0 (0%)	6 (6%)	18 (19%)	23 (25%)	1 (1%)	11 (12%)	7 (8%)
	Poor	0 (0%)	1 (1%)	1 (1%)	2 (2%)	0 (0%)	2 (2%)	0 (0%)	1 (1%)	1 (1%)	6 (6%)	4 (4%)
**HLH**	Excellent	14 (15%)	22 (24%)	28 (30%)	15 (16%)	30 (32%)	26 (28%)	7 (8%)	10 (11%)	61 (66%)	26 (28%)	17 (18%)
	Good	19 (20%)	26 (28%)	26 (28%)	47 (51%)	51 (55%)	21 (23%)	5 (5%)	6 (6%)	22 (24%)	38 (41%)	28 (30%)
	Moderate	44 (47%)	18 (19%)	26 (28%)	30 (32%)	12 (13%)	43 (46%)	49 (53%)	45 (48%)	10 (11%)	27 (29%)	41 (44%)
	Poor	16 (17%)	27 (29%)	12 (13%)	1 (1%)	0 (0%)	3 (3%)	32 (34%)	32 (34%)	0 (0%)	2 (2%)	7 (8%)
**HHL**	Excellent	10 (11%)	40 (43%)	12 (13%)	15 (16%)	20 (22%)	25 (27%)	6 (6%)	8 (9%)	34 (37%)	26 (28%)	10 (11%)
	Good	21 (23%)	22 (24%)	32 (34%)	36 (39%)	43 (46%)	21 (23%)	4 (4%)	4 (4%)	31 (33%)	19 (20%)	10 (11%)
	Moderate	53 (57%)	28 (30%)	39 (42%)	36 (39%)	30 (32%)	34 (37%)	17 (18%)	25 (27%)	24 (26%)	28 (30%)	36 (39%)
	Poor	9 (10%)	3 (3%)	10 (11%)	6 (6%)	0 (0%)	13 (14%)	66 (71%)	56 (60%)	4 (4%)	20 (22%)	37 (40%)

**HHH**	Excellent	10 (11%)	61 (66%)	15 (16%)	10 (11%)	23 (25%)	11 (12%)	16 (17%)	13 (14%)	61 (66%)	52 (56%)	30 (32%)
	Good	14 (15%)	24 (26%)	15 (16%)	15 (16%)	15 (16%)	8 (9%)	8 (9%)	4 (4%)	18 (19%)	11 (12%)	22 (24%)
	Moderate	40 (43%)	7 (8%)	24 (26%)	33 (35%)	18 (19%)	51 (55%)	16 (17%)	19 (20%)	10 (11%)	26 (28%)	23 (25%)
	Poor	29 (31%)	1 (1%)	39 (42%)	35 (38%)	37 (40%)	23 (25%)	53 (57%)	57 (61%)	4 (4%)	4 (4%)	18 (19%)
**LLL**	Excellent	90 (97%)	89 (96%)	90 (97%)	87 (94%)	91 (98%)	91 (98%)	90 (97%)	93 (100%)	90 (97%)	89 (96%)	88 (95%)
	Good	3 (3%)	2 (2%)	2 (2%)	4 (4%)	2 (2%)	2 (2%)	3 (3%)	0 (0%)	3 (3%)	2 (2%)	2 (2%)
	Moderate	0 (0%)	0 (0%)	1 (1%)	2 (2%)	0 (0%)	0 (0%)	0 (0%)	0 (0%)	0 (0%)	0 (0%)	3 (3%)
	Poor	0 (0%)	2 (2%)	0 (0%)	0 (0%)	0 (0%)	0 (0%)	0 (0%)	0 (0%)	0 (0%)	2 (2%)	0 (0%)

Table S3. Distribution of radiomics feature reproducibility scores (ICC-based: Excellent, Good, Moderate, Poor) across 11 organs (aorta, gallbladder, inferior vena cava (IVC), left kidney, right kidney, liver, pancreas, spleen, stomach, portal vein and splenic vein, and soft tissue metastases) and 9 feature groups (Original and eight wavelet-transformed groups) using a bin width of 150.

	**Quality**	**STM**	**PSV**	**Stomach**	**Spleen**	**Pancreas**	**Liver**	**RK**	**LK**	**IVC**	**GB**	**Aorta**
**Original**	Excellent	86 (92%)	89 (96%)	92 (99%)	85 (91%)	90 (97%)	91 (98%)	87 (94%)	89 (96%)	90 (97%)	87 (94%)	89 (96%)
	Good	7 (8%)	3 (3%)	0 (0%)	8 (9%)	2 (2%)	2 (2%)	5 (5%)	3 (3%)	3 (3%)	5 (5%)	4 (4%)
	Moderate	0 (0%)	0 (0%)	1 (1%)	0 (0%)	1 (1%)	0 (0%)	1 (1%)	1 (1%)	0 (0%)	0 (0%)	0 (0%)
	Poor	0 (0%)	1 (1%)	0 (0%)	0 (0%)	0 (0%)	0 (0%)	0 (0%)	0 (0%)	0 (0%)	1 (1%)	0 (0%)
**LLH**	Excellent	33 (35%)	59 (63%)	60 (65%)	43 (46%)	80 (86%)	49 (53%)	20 (22%)	24 (26%)	68 (73%)	62 (67%)	49 (53%)
	Good	47 (51%)	21 (23%)	32 (34%)	38 (41%)	12 (13%)	35 (38%)	54 (58%)	54 (58%)	15 (16%)	23 (25%)	36 (39%)
	Moderate	10 (11%)	10 (11%)	1 (1%)	12 (13%)	0 (0%)	9 (10%)	19 (20%)	12 (13%)	6 (6%)	6 (6%)	7 (8%)
	Poor	3 (3%)	3 (3%)	0 (0%)	0 (0%)	1 (1%)	0 (0%)	0 (0%)	3 (3%)	4 (4%)	2 (2%)	1 (1%)
**LHL**	Excellent	50 (54%)	43 (46%)	62 (67%)	62 (67%)	58 (62%)	62 (67%)	51 (55%)	47 (51%)	72 (77%)	68 (73%)	48 (52%)
	Good	37 (40%)	35 (38%)	19 (20%)	23 (25%)	26 (28%)	25 (27%)	35 (38%)	35 (38%)	18 (19%)	14 (15%)	27 (29%)
	Moderate	5 (5%)	14 (15%)	11 (12%)	7 (8%)	8 (9%)	5 (5%)	5 (5%)	10 (11%)	3 (3%)	9 (9%)	15 (16%)
	Poor	1 (1%)	1 (1%)	1 (1%)	1 (1%)	1 (1%)	1 (1%)	2 (2%)	1 (1%)	0 (0%)	3 (3%)	3 (3%)
**LHH**	Excellent	19 (20%)	69 (74%)	16 (17%)	16 (17%)	22 (24%)	21 (23%)	11 (12%)	11 (12%)	63 (68%)	50 (54%)	19 (20%)
	Good	33 (35%)	18 (19%)	32 (34%)	27 (29%)	24 (26%)	26 (28%)	6 (6%)	6 (6%)	24 (26%)	28 (30%)	21 (23%)
	Moderate	36 (39%)	5 (5%)	41 (44%)	40 (43%)	46 (49%)	30 (32%)	29 (31%)	31 (33%)	5 (5%)	14 (15%)	44 (47%)
	Poor	5 (5%)	1 (1%)	4 (4%)	10 (11%)	1 (1%)	16 (17%)	47 (51%)	45 (48%)	1 (1%)	1 (1%)	9 (10%)
**HLL**	Excellent	42 (45%)	74 (80%)	61 (66%)	62 (67%)	78 (84%)	63 (68%)	35 (38%)	36 (39%)	70 (75%)	49 (53%)	48 (52%)
	Good	45 (48%)	12 (13%)	25 (27%)	23 (25%)	15 (16%)	28 (30%)	40 (43%)	36 (39%)	20 (22%)	24 (26%)	36 (39%)
	Moderate	4 (4%)	5 (5%)	6 (6%)	4 (4%)	0 (0%)	2 (2%)	18 (19%)	19 (20%)	2 (2%)	14 (14%)	9 (9%)
	Poor	2 (2%)	2 (2%)	1 (1%)	4 (4%)	0 (0%)	0 (0%)	0 (0%)	2 (2%)	1 (1%)	8 (8%)	1 (1%)
**HLH**	Excellent	16 (17%)	26 (28%)	24 (26%)	14 (15%)	27 (29%)	24 (26%)	7 (8%)	9 (10%)	61 (66%)	23 (25%)	13 (14%)
	Good	20 (22%)	21 (23%)	29 (31%)	25 (27%)	49 (53%)	21 (23%)	7 (8%)	9 (10%)	19 (20%)	34 (37%)	27 (29%)
	Moderate	48 (52%)	17 (18%)	32 (34%)	49 (53%)	17 (18%)	37 (40%)	38 (41%)	42 (45%)	12 (13%)	31 (33%)	48 (52%)
	Poor	9 (10%)	29 (31%)	8 (9%)	5 (5%)	0 (0%)	11 (12%)	41 (44%)	34 (37%)	1 (1%)	5 (5%)	5 (5%)

HHL	Excellent	13 (14%)	51 (55%)	12 (13%)	11 (12%)	29 (31%)	12 (13%)	6 (6%)	9 (10%)	35 (38%)	25 (27%)	25 (27%)
	Good	26 (28%)	18 (19%)	25 (27%)	30 (32%)	46 (49%)	22 (24%)	5 (5%)	4 (4%)	30 (32%)	16 (17%)	14 (15%)
	Moderate	46 (49%)	10 (11%)	45 (48%)	46 (49%)	18 (19%)	42 (45%)	15 (16%)	14 (15%)	24 (26%)	29 (31%)	35 (38%)
	Poor	8 (9%)	14 (15%)	11 (12%)	6 (6%)	0 (0%)	17 (18%)	67 (72%)	66 (71%)	4 (4%)	23 (25%)	19 (20%)
HHH	Excellent	12 (13%)	61 (66%)	21 (23%)	18 (19%)	24 (26%)	9 (10%)	22 (24%)	13 (14%)	64 (69%)	57 (61%)	30 (32%)
	Good	16 (17%)	24 (26%)	20 (22%)	14 (15%)	16 (17%)	9 (10%)	10 (11%)	13 (14%)	17 (18%)	7 (8%)	22 (24%)
	Moderate	31 (33%)	7 (8%)	33 (35%)	20 (22%)	26 (28%)	51 (55%)	14 (15%)	27 (29%)	12 (13%)	18 (19%)	23 (25%)
	Poor	34 (37%)	1 (1%)	19 (20%)	41 (44%)	27 (29%)	24 (26%)	47 (51%)	40 (43%)	0 (0%)	11 (12%)	18 (19%)
LLL	Excellent	84 (90%)	68 (73%)	86 (92%)	89 (97%)	92 (99%)	91 (98%)	92 (99%)	90 (97%)	89 (97%)	90 (97%)	90 (97%)
	Good	9 (10%)	14 (15%)	4 (4%)	4 (4%)	1 (1%)	2 (1%)	1 (1%)	3 (3%)	4 (4%)	1 (1%)	2 (2%)
	Moderate	0 (0%)	7 (8%)	3 (3%)	0 (0%)	0 (0%)	0 (0%)	0 (0%)	0 (0%)	0 (0%)	0 (0%)	0 (0%)
	Poor	0 (0%)	4 (4%)	0 (0%)	0 (0%)	0 (0%)	0 (0%)	0 (0%)	0 (0%)	0 (0%)	2 (2%)	1 (1%)

Table S4. Distribution of radiomics feature reproducibility scores (ICC-based: Excellent, Good, Moderate, Poor) across 11 organs (aorta, gallbladder, inferior vena cava (IVC), left kidney, right kidney, liver, pancreas, spleen, stomach, portal vein and splenic vein, and soft tissue metastases) and 9 feature groups (Original and eight wavelet-transformed groups) using a bin width of 200.

	Quality	STM	PSV	Stomach	Spleen	Pancreas	Liver	RK	LK	IVC	GB	Aorta
Original	Excellent	84 (90%)	91 (98%)	90 (97%)	91 (98%)	91 (98%)	91 (98%)	91 (98%)	89 (96%)	93 (100%)	90 (97%)	85 (91%)
	Good	7 (8%)	1 (1%)	1 (1%)	2 (2%)	1 (1%)	2 (2%)	2 (2%)	4 (4%)	0 (0%)	2 (2%)	7 (8%)
	Moderate	2 (2%)	0 (0%)	2 (2%)	0 (0%)	1 (1%)	0 (0%)	0 (0%)	0 (0%)	0 (0%)	0 (0%)	0 (0%)
	Poor	0 (0%)	1 (1%)	0 (0%)	0 (0%)	0 (0%)	0 (0%)	0 (0%)	0 (0%)	0 (0%)	1 (1%)	1 (1%)
LLH	Excellent	31 (33%)	58 (62%)	64 (69%)	43 (46%)	82 (88%)	44 (47%)	18 (19%)	24 (26%)	84 (90%)	62 (67%)	44 (47%)
	Good	49 (53%)	24 (26%)	24 (26%)	32 (34%)	8 (9%)	37 (40%)	53 (57%)	46 (49%)	9 (10%)	19 (20%)	38 (41%)
	Moderate	10 (11%)	8 (9%)	4 (4%)	14 (15%)	2 (2%)	11 (12%)	21 (23%)	22 (24%)	0 (0%)	9 (10%)	10 (11%)
	Poor	3 (3%)	3 (3%)	1 (1%)	4 (4%)	1 (1%)	1 (1%)	1 (1%)	1 (1%)	0 (0%)	3 (3%)	1 (1%)
LHL	Excellent	53 (57%)	51 (55%)	62 (67%)	65 (70%)	65 (70%)	63 (68%)	53 (57%)	46 (49%)	67 (72%)	69 (74%)	40 (43%)
	Good	34 (37%)	30 (32%)	16 (17%)	18 (19%)	21 (23%)	23 (25%)	27 (29%)	37 (40%)	20 (22%)	21 (23%)	29 (31%)
	Moderate	5 (5%)	11 (12%)	13 (14%)	6 (6%)	7 (8%)	7 (8%)	12 (13%)	9 (10%)	6 (6%)	1 (1%)	21 (23%)
	Poor	1 (1%)	1 (1%)	2 (2%)	4 (4%)	0 (0%)	0 (0%)	1 (1%)	1 (1%)	0 (0%)	2 (2%)	3 (3%)
LHH	Excellent	28 (30%)	68 (73%)	16 (17%)	17 (18%)	22 (24%)	12 (13%)	10 (11%)	12 (13%)	61 (66%)	51 (55%)	18 (19%)
	Good	28 (30%)	19 (20%)	31 (33%)	22 (24%)	16 (17%)	30 (32%)	5 (5%)	4 (4%)	26 (28%)	22 (24%)	25 (27%)
	Moderate	31 (33%)	5 (5%)	41 (44%)	29 (31%)	42 (45%)	37 (40%)	36 (39%)	33 (35%)	5 (5%)	19 (20%)	41 (44%)
	Poor	6 (6%)	1 (1%)	5 (5%)	25 (27%)	13 (14%)	14 (15%)	42 (45%)	44 (47%)	1 (1%)	1 (1%)	9 (10%)
HLL	Excellent	47 (51%)	80 (86%)	63 (68%)	59 (63%)	74 (80%)	55 (59%)	37 (40%)	34 (37%)	67 (72%)	69 (74%)	43 (46%)
	Good	40 (43%)	10 (11%)	23 (25%)	20 (22%)	17 (18%)	25 (27%)	36 (39%)	38 (41%)	23 (25%)	18 (19%)	36 (39%)
	Moderate	5 (5%)	2 (2%)	6 (6%)	10 (11%)	2 (2%)	10 (11%)	20 (22%)	17 (18%)	2 (2%)	4 (4%)	13 (14%)
	Poor	1 (1%)	1 (1%)	1 (1%)	4 (4%)	0 (0%)	3 (3%)	0 (0%)	4 (4%)	1 (1%)	2 (2%)	1 (1%)

**HLH**	Excellent	16 (17%)	24 (26%)	13 (14%)	12 (13%)	25 (27%)	24 (26%)	8 (9%)	13 (14%)	48 (52%)	31 (33%)	12 (13%)
	Good	21 (23%)	22 (24%)	30 (32%)	21 (23%)	49 (53%)	17 (18%)	10 (11%)	6 (6%)	17 (18%)	23 (25%)	18 (19%)
	Moderate	44 (47%)	18 (19%)	40 (43%)	54 (58%)	19 (20%)	38 (41%)	32 (34%)	39 (42%)	24 (26%)	32 (34%)	42 (45%)
	Poor	12 (13%)	29 (31%)	10 (11%)	6 (6%)	0 (0%)	14 (15%)	43 (46%)	35 (38%)	4 (4%)	7 (8%)	21 (23%)
**HHL**	Excellent	17 (18%)	70 (75%)	12 (13%)	12 (13%)	28 (30%)	11 (12%)	8 (9%)	10 (11%)	36 (39%)	26 (28%)	11 (12%)
	Good	19 (20%)	15 (16%)	31 (33%)	28 (30%)	31 (33%)	21 (23%)	4 (4%)	3 (3%)	34 (37%)	16 (17%)	14 (15%)
	Moderate	51 (55%)	7 (8%)	37 (40%)	45 (48%)	31 (33%)	45 (48%)	16 (17%)	16 (17%)	20 (22%)	24 (26%)	40 (43%)
	Poor	6 (6%)	1 (1%)	13 (14%)	8 (9%)	3 (3%)	16 (17%)	65 (70%)	64 (69%)	3 (3%)	27 (29%)	28 (30%)
**HHH**	Excellent	14 (15%)	59 (63%)	22 (24%)	22 (24%)	25 (27%)	8 (9%)	26 (28%)	16 (17%)	63 (68%)	61 (66%)	30 (32%)
	Good	12 (13%)	26 (28%)	20 (22%)	15 (16%)	18 (19%)	11 (12%)	22 (24%)	30 (32%)	18 (19%)	3 (3%)	23 (25%)
	Moderate	32 (34%)	7 (8%)	45 (48%)	17 (18%)	26 (28%)	49 (53%)	30 (32%)	25 (27%)	12 (13%)	17 (18%)	22 (24%)
	Poor	35 (38%)	1 (1%)	6 (6%)	39 (42%)	24 (26%)	25 (27%)	15 (16%)	22 (24%)	0 (0%)	12 (13%)	18 (19%)
**LLL**	Excellent	90 (97%)	67 (72%)	91 (98%)	84 (90%)	91 (98%)	93 (100%)	93 (100%)	91 (98%)	91 (98%)	89 (96%)	89 (96%)
	Good	3 (3%)	15 (16%)	1 (1%)	7 (8%)	2 (1%)	0 (0%)	0 (0%)	2 (2%)	2 (2%)	2 (2%)	2 (2%)
	Moderate	0 (0%)	8 (9%)	1 (1%)	2 (1%)	0 (0%)	0 (0%)	0 (0%)	0 (0%)	0 (0%)	0 (0%)	2 (2%)
	Poor	0 (0%)	3 (3%)	0 (0%)	0 (0%)	0 (0%)	0 (0%)	0 (0%)	0 (0%)	0 (0%)	2 (2%)	0 (0%)

Table S5. Organ-wise distribution of radiomic features across concordance correlation coefficient (CCC) agreement categories (Perfect, Substantial, Moderate, Poor) for three pairwise reconstruction comparisons at four discretization bin widths (BW = 50, 100, 150, 200). For each organ and CCC category, values are reported as RL–RVC|RL–SPECT|RVC–SPECTRL–RVC | RL–SPECT | RVC–SPECTRL–RVC|RL–SPECT|RVC–SPECT, representing the number of radiomic features falling within the corresponding agreement range (Perfect ≥ 0.95, Substantial 0.90–0.95, Moderate 0.80–0.90, Poor < 0.80).

<table>
<tr><th></th><th>Aorta</th><th>Gallbladder</th><th>Inferion vena cava</th><th>Left kidney</th><th>Right kidney</th><th>Liver</th><th>Pancreas</th><th>Spleen</th><th>Stomach</th><th>Portal vein and splenic vein</th><th>Soft tissue metastasis</th></tr>
<tr><td>Perfect-BW 50</td><td>[484|109|135]</td><td>[462|105|127]</td><td>[490|170|172]</td><td>[452|130|145]</td><td>[542|153|162]</td><td>[475|122|138]</td><td>[426|72|90]</td><td>[430|93|105]</td><td>[511|145|9]</td><td>[458|147|161]</td><td>[410|131|140]</td></tr>
<tr><td>Perfect-BW 100</td><td>[387|47|60]</td><td>[431|93|128]</td><td>[455|172|182]</td><td>[430|103|114]</td><td>[503|126|136]</td><td>[462|128|137]</td><td>[410|68|81]</td><td>[424|80|88]</td><td>[544|169|192]</td><td>[470|111|129]</td><td>[313|119|115]</td></tr>
<tr><td>Perfect-BW 150</td><td>[416|46|53]</td><td>[386|111|146]</td><td>[440|172|182]</td><td>[433|103|112]</td><td>[535|129|142]</td><td>[460|130|137]</td><td>[409|68|78]</td><td>[407|76|85]</td><td>[525|156|187]</td><td>[452|121|144]</td><td>[308|135|141]</td></tr>
<tr><td>Perfect-BW 200</td><td>[408|46|58]</td><td>[411|116|143]</td><td>[471|181|190]</td><td>[423|103|116]</td><td>[493|134|146]</td><td>[456|124|139]</td><td>[398|74|82]</td><td>[401|78|89]</td><td>[510|157|184]</td><td>[516|145|182]</td><td>[298|113|126]</td></tr>
<tr><td>Substantial-BW 50</td><td>[165|189|209]</td><td>[183|167|201]</td><td>[186|120|131]</td><td>[187|129|156]</td><td>[158|139|154]</td><td>[185|126|151]</td><td>[191|178|195]</td><td>[182|163|181]</td><td>[180|183|211]</td><td>[202|176|188]</td><td>[239|138|154]</td></tr>
<tr><td>Substantial-BW 100</td><td>[206|95|119]</td><td>[186|175|261]</td><td>[145|114|127]</td><td>[147|127|160]</td><td>[163|158|188]</td><td>[158|117|149]</td><td>[110|138|157]</td><td>[118|136|153]</td><td>[180|239|262]</td><td>[182|192|227]</td><td>[185|124|158]</td></tr>
<tr><td>Substantial-BW 150</td><td>[192|75|105]</td><td>[204|167|273]</td><td>[141|101|112]</td><td>[134|135|163]</td><td>[160|146|181]</td><td>[140|114|141]</td><td>[109|133|156]</td><td>[107|132|148]</td><td>[202|213|247]</td><td>[161|197|267]</td><td>[191|126|150]</td></tr>
<tr><td>Substantial-BW 200</td><td>[181|86|115]</td><td>[212|201|313]</td><td>[146|103|120]</td><td>[122|131|181]</td><td>[153|144|174]</td><td>[135|119|136]</td><td>[105|129|156]</td><td>[118|125|139]</td><td>[202|216|255]</td><td>[164|223|250]</td><td>[167|114|154]</td></tr>
<tr><td>Moderate-BW 50</td><td>[145|231|234]</td><td>[145|308|304]</td><td>[123|209|257]</td><td>[131|229|259]</td><td>[99|278|293]</td><td>[122|222|241]</td><td>[138|168|175]</td><td>[146|170|172]</td><td>[116|258|280]</td><td>[130|220|239]</td><td>[139|233|228]</td></tr>
</table>

**Moderate-BW 100**	[163|263|305]	[139|307|275]	[155|190|253]	[178|213|263]	[103|258|283]	[139|219|223]	[146|153|171]	[131|152|163]	[83|238|254]	[137|228|224]	[212|208|227]
**Moderate-BW 150**	[159|284|312]	[148|311|251]	[182|204|260]	[178|204|249]	[86|252|286]	[138|219|239]	[150|168|201]	[142|163|189]	[86|284|267]	[125|227|195]	[204|223|245]
**Moderate-BW 200**	[160|285|327]	[136|299|232]	[147|172|225]	[188|206|234]	[111|251|283]	[147|188|216]	[155|169|209]	[140|170|203]	[87|275|271]	[104|205|194]	[236|205|218]
**Poor-BW 50**	[43|308|259]	[38|248|196]	[38|338|276]	[67|349|277]	[38|267|228]	[55|367|307]	[82|419|377]	[79|411|379]	[30|251|194]	[38|285|240]	[49|335|315]
**Poor-BW 100**	[81|432|353]	[72|253|164]	[82|361|275]	[82|394|300]	[68|295|230]	[78|373|328]	[171|478|428]	[164|469|433]	[30|191|129]	[38|297|249]	[127|386|337]
**Poor-BW 150**	[70|432|367]	[90|239|158]	[74|360|283]	[92|395|312]	[56|310|228]	[99|374|320]	[169|468|402]	[181|466|415]	[24|184|136]	[90|283|220]	[134|353|301]
**Poor-BW 200**	[88|420|337]	[69|212|140]	[73|381|302]	[103|396|305]	[80|308|234]	[99|406|346]	[179|465|390]	[178|464|406]	[38|189|127]	[43|255|202]	[136|405|339]
